# The Efficacy Comparison of Endoscopic Bariatric Therapies: 6-Month Versus 12-Month Intragastric Balloon Versus Endoscopic Sleeve Gastroplasty

**DOI:** 10.1007/s11695-022-06398-x

**Published:** 2022-12-16

**Authors:** K. Kozłowska-Petriczko, K. M. Pawlak, K. Wojciechowska, A. Reiter, Ł. Błaszczyk, J. Szełemej, J. Petriczko, A. Wiechowska-Kozłowska

**Affiliations:** 1 Translational Medicine Group, Pomeranian Medical University, Szczecin, Poland; 2 Endoscopy Unit, Department of Gastroenterology, Hospital of the Ministry of Interior and Administration, Szczecin, Poland; 3 Department of Internal Medicine and Gastroenterology, Pomeranian Medical University, Szczecin, Poland; 4 Endoscopy Unit, Regional Health Center, Lubin, Poland; 5 Endoscopy Unit, Sonomed Medical Centre, Szczecin, Poland; 6 Department of Plastic, Endocrine and General Surgery, Pomeranian Medical University, Szczecin, Poland

**Keywords:** Obesity management, Endoscopic bariatric therapy, Intragastric balloon, Endoscopic sleeve gastroplasty, Orbera 365

## Abstract

**Introduction:**

Intragastric balloon (IGB) insertion and endoscopic sleeve gastroplasty (ESG) are known to be effective and safe in achieving weight loss. The aim of this study was to compare the effects of a 6-month IGB therapy, a 12-month IGB therapy, and ESG.

**Methods:**

We retrospectively analyzed the weight loss at IGB (Orbera) removal after 6 months (124 patients), at IGB (Orbera365) removal after 12 months (61 patients) and at 6 and 12 months after ESG (42 and 34 patients, respectively). Postprocedural care, including medication and diet, was the same for all procedures.

**Results:**

Mean TBWL in patients undergoing IGB placement for 6 and 12 months and ESG after 6 and 12 months were 15.2, 15.8, 26.5, and 28.7 kg, respectively. There was no significant difference in the mean %TBWL in patients undergoing IGB placement for 6 or for 12 months (15.3% vs. 14.7%, *P* = 0.7). ESG patients showed a significantly higher mean %TBWL than IGB patients after 6 months (15.3 vs. 19.8, *P* = 0.005) and 12 months (14.7 vs. 22.5, *P* < 0.001).

**Conclusion:**

All three studied methods were effective for achieving weight loss. However, there was no significant difference between 6-month and 12-month IGB therapies outcomes. ESG appeared to be a more effective obesity treatment modality than IGB.

**Graphical Abstract:**

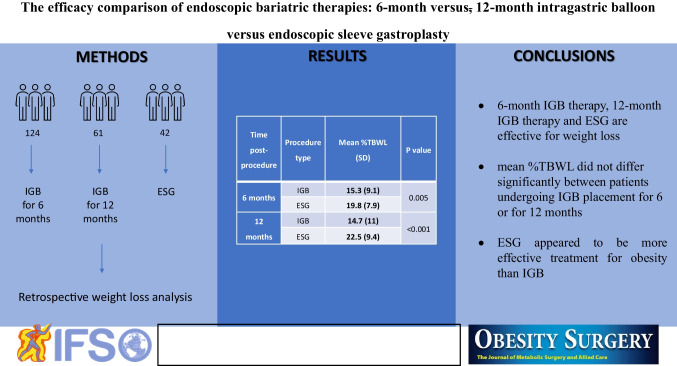

## Introduction

Overweight and obesity currently constitute a real global public health burden. Their prevalence worldwide is high and constantly rising, currently estimated at 39% adult world population. It is not only a serious condition limiting the quality of life but also a chronic disease predisposing to numerous complications [1].

Lifestyle changes, pharmacotherapy, and diet, although widely available, seem to be insufficient for the majority of patients. At most, a 5–10% total body weight loss (TBWL) can be achieved in 50% of cases, assuming strict patient cooperation [2, 3]. Among all procedural treatment options, bariatric surgery remains the most effective method for durable weight loss, however due to its invasiveness and the risk of early or late postoperative complications, endoscopic bariatric therapies (EBTs) may bridge the gap between conservative and surgical treatment, particularly for individuals who are not eligible for or are not willing to undergo surgery. EBTs provide minimally invasive, reversible, and repeatable treatment for patients with obesity [4–6]. Numerous endoscopic bariatric techniques are now available, allowing a more personalized and tailored approach to the patient and their comorbidities [4].

Among all EBTs, intragastric balloons (IGBs) as space-occupying devices are considered the most popular and widely available. Their mechanism of action is multifaceted, involving physiological and neurohormonal changes. The balloons function as an artificial bezoar filling the stomach, promoting early satiety [3, 7]. There are many different IGB types on the market, three of which have been approved by the FDA [4]: the Orbera intragastric balloon (Apollo Endosurgery, Austin, TX), the ReShape Duo (ReShape Medical, San Clemente, CA), and the Obalon IGB (Obalon Therapeutics, Carlsbad, CA). All these models are placed into the stomach for a period of 6 months, and have well-established efficacy [3]. A meta-analysis published by the American Society for Gastrointestinal Endoscopy (ASGE) depicted that Orbera IGB resulted in an overall %TBWL of 13.16% after 6 months [8]. On top of that, the recently published meta-analysis, including 2013 patients with a BMI ranging from 30.6 to 36.2 kg/m^2^, showed a %TBWL of 12.8% after completion of treatment (at 4–6 months) and a %TBWL of 10.9% at 1-year follow-up [5]. The Orbera365 is a relatively new balloon, designed for 12 months of therapy. However, there is little data on its efficacy [9] and no data comparing different dwelling times.

IGBs, although popular, may not be sufficient to achieve long-term weight loss due to poor tolerability estimated at about 93% and weight recidivism following balloon removal [4]. On the other hand, endoscopic sleeve gastroplasty (ESG) has achieved significant success in the past few years as a promising minimally invasive, cost-effective, and reversible treatment in mild to moderate obesity [10, 11]. The procedure relies on gastric wall plication using an endoscopic suturing device (OverStitch, Apollo Endosurgery, Austin, TX) which changes the anatomy and physiology of the stomach by reducing its volume to approximately 80%. The safety and efficacy of ESG have recently been confirmed by meta-analyses showing TBWL at 12 months of 16.1–16.5%, with a serious adverse event rate in the range of 2.2 to 2.3%.

In this retrospective study, we compared the efficacy of IGB therapies with dwelling time of 6 months versus 12 months. Additionally, we compared both these methods to ESG.

## Materials and Methods

### Patient Population

We retrospectively identified 246 patients who underwent EBTs between 2015 and 2020 in two centers located in Poland (Sonomed Medical Center, Szczecin, Poland and Sokolowski Hospital, Walbrzych, Poland). The Institutional Ethics Committee approved the study (IRB 3857/2022P). All procedures were performed following the ethical principles detailed in the Declaration of Helsinki.

All patients had initial consultations provided by dietitian, psychologist, and gastroenterologist in order to deliver comprehensive.

information on various weight loss therapies including diet and exercise programs, pharmacotherapy, EBT, and bariatric surgery. Endoscopic procedures included ESG or IGB insertion for either 6 or 12 months and were offered to patients unsuccessfully treated with conservative methods (diet and/or exercise and/or medications) for at least 3 months, who were not eligible for or elected not to undergo bariatric surgery. Further inclusion criteria were BMI over 27 kg/m^2^ for IGB and BMI over 30 kg/m^2^ for ESG. General contraindications to both procedures included previous gastroesophageal surgery, ongoing anticoagulation, active gastric ulceration, decompensated organ failure, pregnancy, or lactation; additional exclusion criteria have been implemented for ESG including personal or family history of gastric malignancy, the presence of any gastric condition requiring endoscopic surveillance (e.g., known gastric intestinal metaplasia), and vascular abnormalities. The technicality of all endoscopic treatment options, risks, benefits, cost, and adverse events were discussed. The final choice of therapy was made by the patient, unless a contraindication existed.

**Fig. 1:**
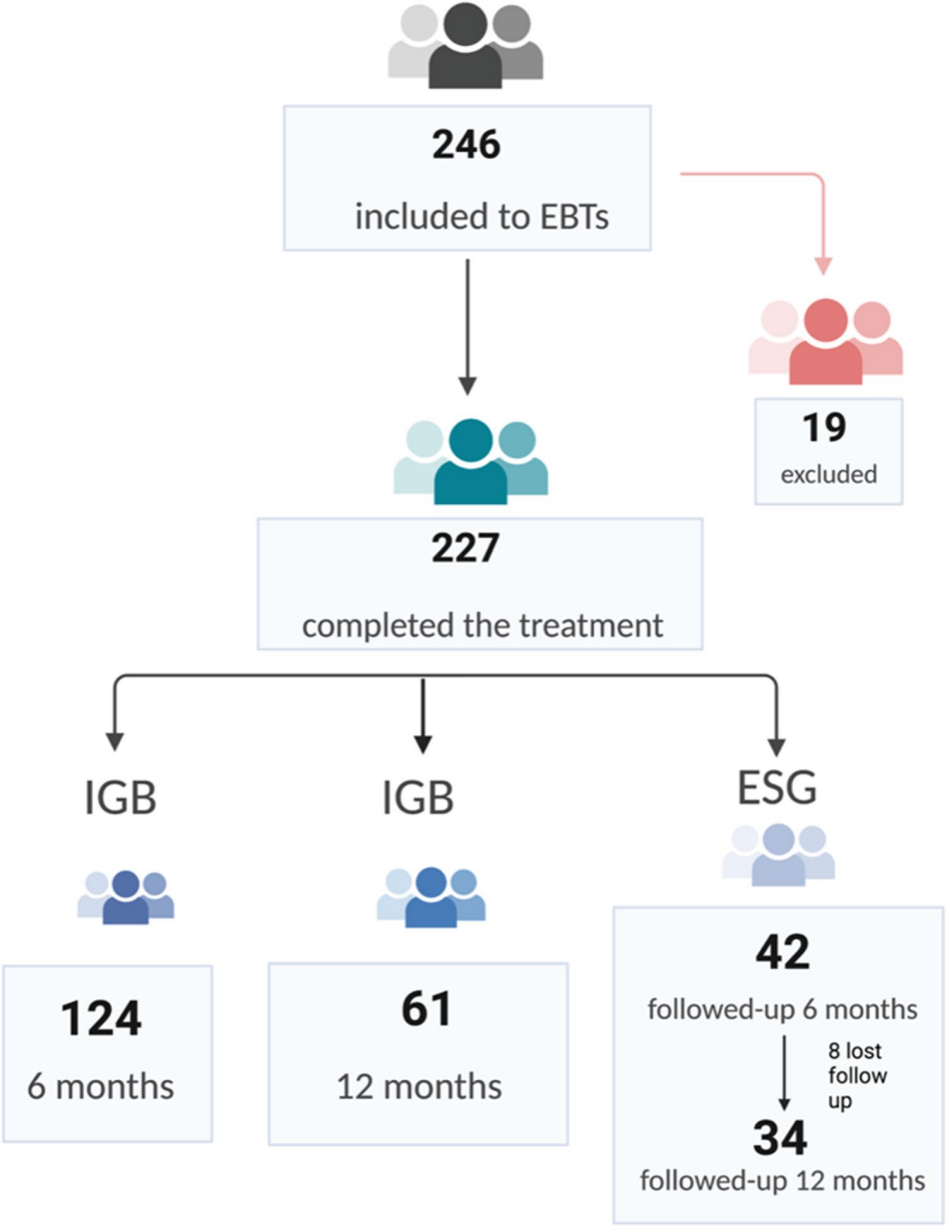
The study flow chart

A total of 227 patients were included in the final data analysis. 124 patients underwent IGB insertion for 6 months, 61 underwent IGB insertion for 12 months and 42 underwent ESG (Fig. 1). We excluded 19 patients who underwent early IGB removal due to intolerance.

### Peri-procedural and Post-procedural Care

All IGB procedures were performed by the same endoscopist (A.W.) and all ESG procedures were performed by the other endoscopist experienced in endoscopic suturing (A.R.). Pre- and post-procedural care, including medication and diet was identical for all procedures. All procedures were performed under general anesthesia. An esophagogastroduodenoscopy had been done routinely in all cases in order to exclude disqualifying gastric or duodenal pathologies of the mucosa.

The preprocedural medications included omeprazole 40 mg for 1 week orally and a single dose of simethicone 1 h before the procedure. The patients were required to fast 8 h before the procedure and were put on a liquid diet from 1 day.

Postoperatively, patients received 2 L of fluids intravenously with 8 mg of ondansetron 80 mg of drotaverine during their stay in the endoscopy unit. All patients were prescribed on-demand medications to prevent adverse events (ondansetron 4 mg for nausea, drotaverine 40 mg for cramping, omeprazole 20 mg daily (40 mg as needed for GERD symptoms)).

The postoperative diet regimen included a strict liquid diet for 3 days, followed by one week of pureed, then soft and ultimately solid diet, if well tolerated. Diet instructions were the same for all patients. During the entire treatment period, patients could consult a dietitian, psychologist, or gastroenterologist.

### Procedures

#### Intragastric Balloon (IGB) Insertion

**Fig. 2:**
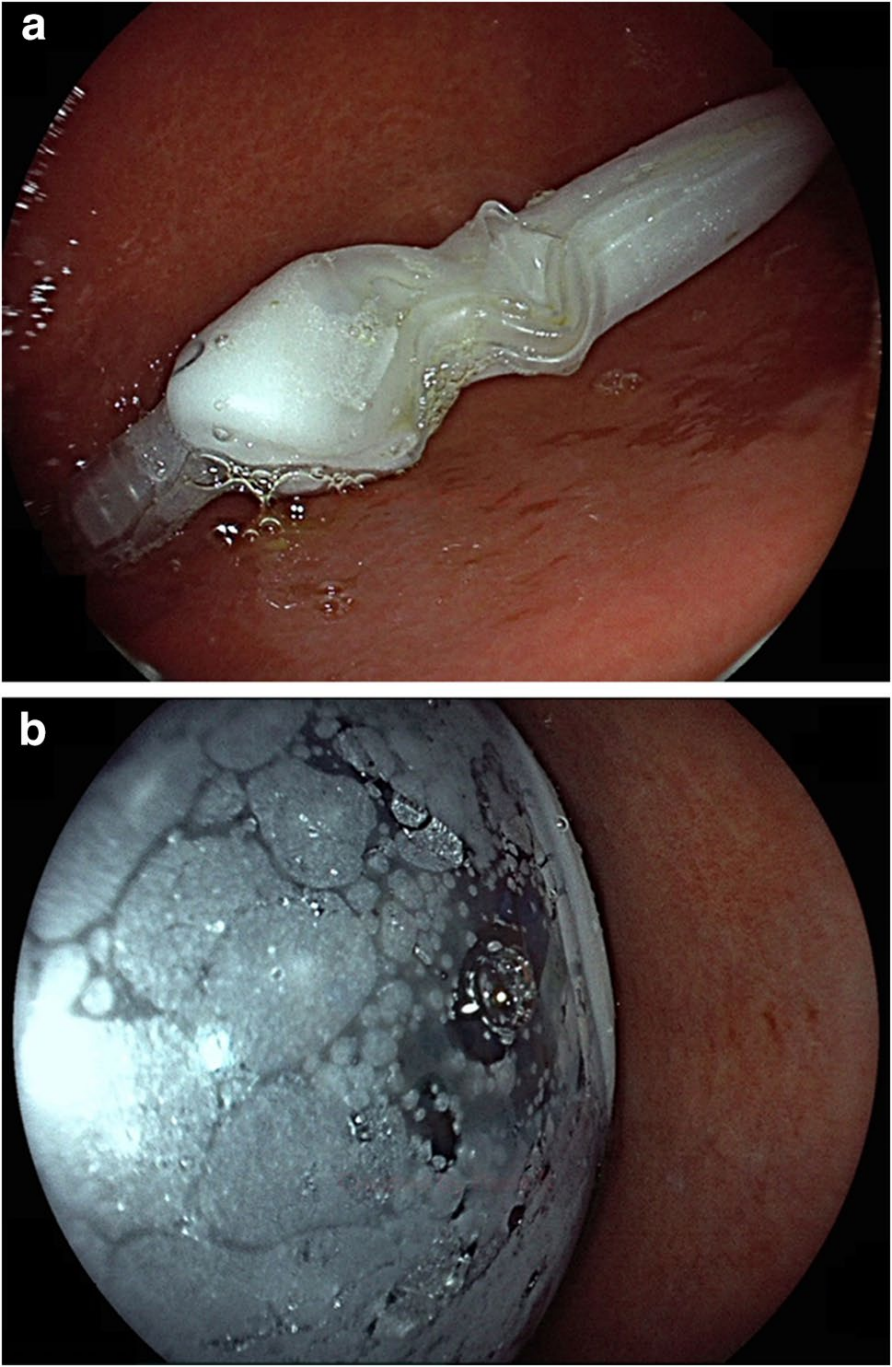
Endoscopic images of Orbera balloon: **a** before inflation, **b** after inflation

Two types of Orbera balloons (Orbera and Orbera365) were inserted endoscopically in line with manufacturer’s recommendations. The balloons were filled with 600–650 ml of 0.9% sodium chloride dyed with methylene blue (Fig. 2). Balloons were removed after 6 or 12 months.

#### Endoscopic Sleeve Gastroplasty (ESG) Procedure

**Fig. 3:**
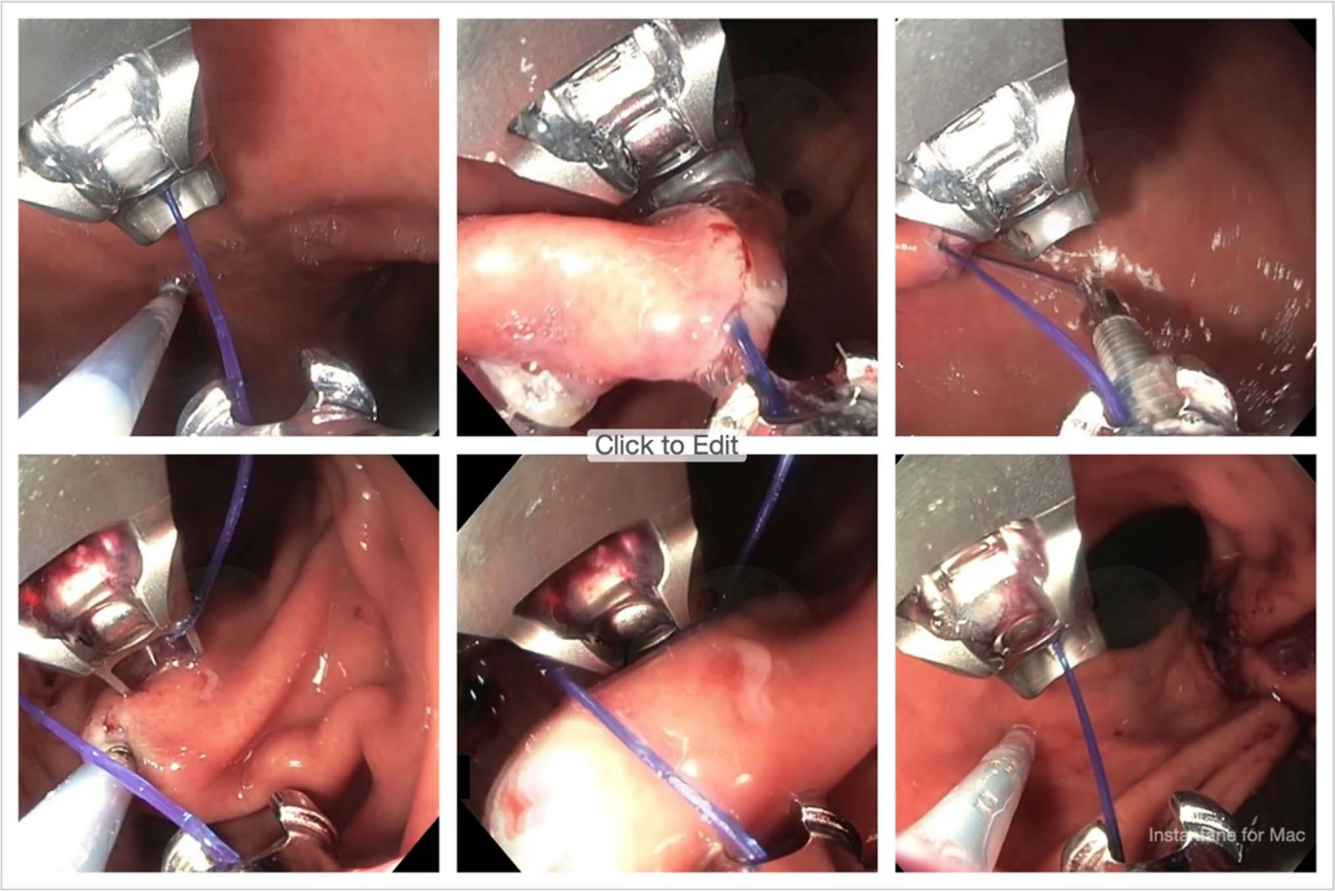
Endoscopic images of ESG procedure. Full-thickness sutures placement from the anterior to the posterior gastric wall along the greater curvature

All ESG procedures have been conducted in the same manner using a two-channel endoscope equipped with a disposable suture device (OverStitch, Apollo Endosurgery, Austin, TX), introduced through the overtube. After identification of the anatomical regions of the stomach, 4 to 7 full-thickness sutures have been placed starting distally to constrict the gastric lumen into a tubular configuration. Each suture comprised six to seven bites from the anterior to the posterior gastric wall along the greater curvature (Fig. 3).

### Statistical Analysis

All statistical analyses were performed using the STATA 11 software (StataCorp, College Station, TX, USA). The Kolmogorov–Smirnov test was used to assess the distribution of variables. Continuous variables are shown as mean ± standard deviation (SD). Parametric test (Student’s *t*-test) was used for the assessment of procedure efficacy. Pearson’s correlation coefficient was used to assess correlations between continuous variables. A three-way analysis of variance (ANOVA) was used to evaluate the impact of age, initial BMI, and procedure type on TBWL. A *P* value of < 0.05 was considered significant.

## Results

Table 1 shows the general characteristics of all patients. The mean age was similar in all groups. The baseline BMI was significantly higher in patients undergoing ESG compared with those undergoing IGB placement, regardless of treatment time—6 or 12 months (44.8 kg/m^2^ vs. 34.2 kg/m^2^ or 36.7 kg/m^2^, *P* < 0.001). The majority of patients were female (69.5%, 83.9%, and 67.21%, respectively).

**Table 1:** Baseline characteristics of the 227 patients who underwent either endoscopic sleeve gastrectomy (ESG) or intragastric balloon (IGB) insertion

	IGB 6 months (*n* = 124)	IGB 12 months (*n* = 61)	ESG (*n* = 42)	*P* value
Age, mean (SD), years	42.0 (9.9)	42.0 (11.0)	43.8 (8.8)	0.6
BMI, mean (SD), kg/m^2^	34.2 (5.0)	36.7 (6.7)	44.8 (4.5)	< 0.001
Sex, female, *n* (%)	104 (83.9)	41 (67.21)	29 (69.05)	< 0.05

*BMI*, body mass index; *SD*, standard deviation

**Fig. 4:**
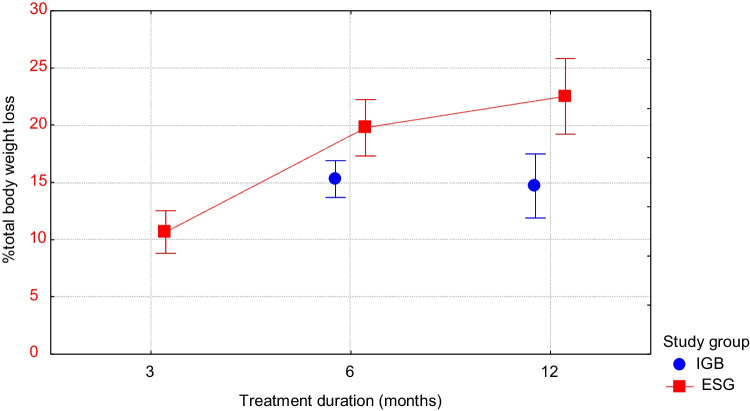
Percentage total body weight loss (%TBWL) at 3, 6, and 12 months after endoscopic sleeve gastrectomy (ESG) or at 6 and 12 months after intragastric balloon (IGB) placement

Table 2 and Fig. 4 show the mean %TBWL at each point in time compared with the baseline. Mean TBWL in patients undergoing IGB placement for 6 and 12 months and ESG after 6 and 12 months were 15.2, 15.8, 26.5, and 28.7 kg, respectively. There was no significant difference in the mean %TBWL in patients undergoing IGB placement for 6 or for 12 months (15.3% vs. 14.7%, *P* = 0.7). ESG patients showed a significantly higher mean %TBWL than IGB patients after 6 months (15.3 vs. 19.8, *P* = 0.005) and 12 months (14.7 vs. 22.5, *P* < 0.001). Between months 6 and 12 of therapy, patients in the ESG group on average lost 4.5 kg in weight.

**Table 2:** Comparison of endoscopic sleeve gastroplasty and intragastric balloon weight loss outcomes

Time post-procedure	Procedure type	Patients (*n*)	Mean TBWL (SD)	Mean %TBWL(SD)	*P* value
6 months	IGB	124	15.2 (9.7)	15.3 (9.1)	0.005
	ESG	42	26.5 (14.3)	19.8 (7.9)	
12 months	IGB	61	15.8 (12.3)	14.7 (11)	< 0.001
	ESG	34	28.7 (12.7)	22.5 (9.4)	

*SD*, standard deviations; *TBWL*, total body weight loss

**Fig. 5:**
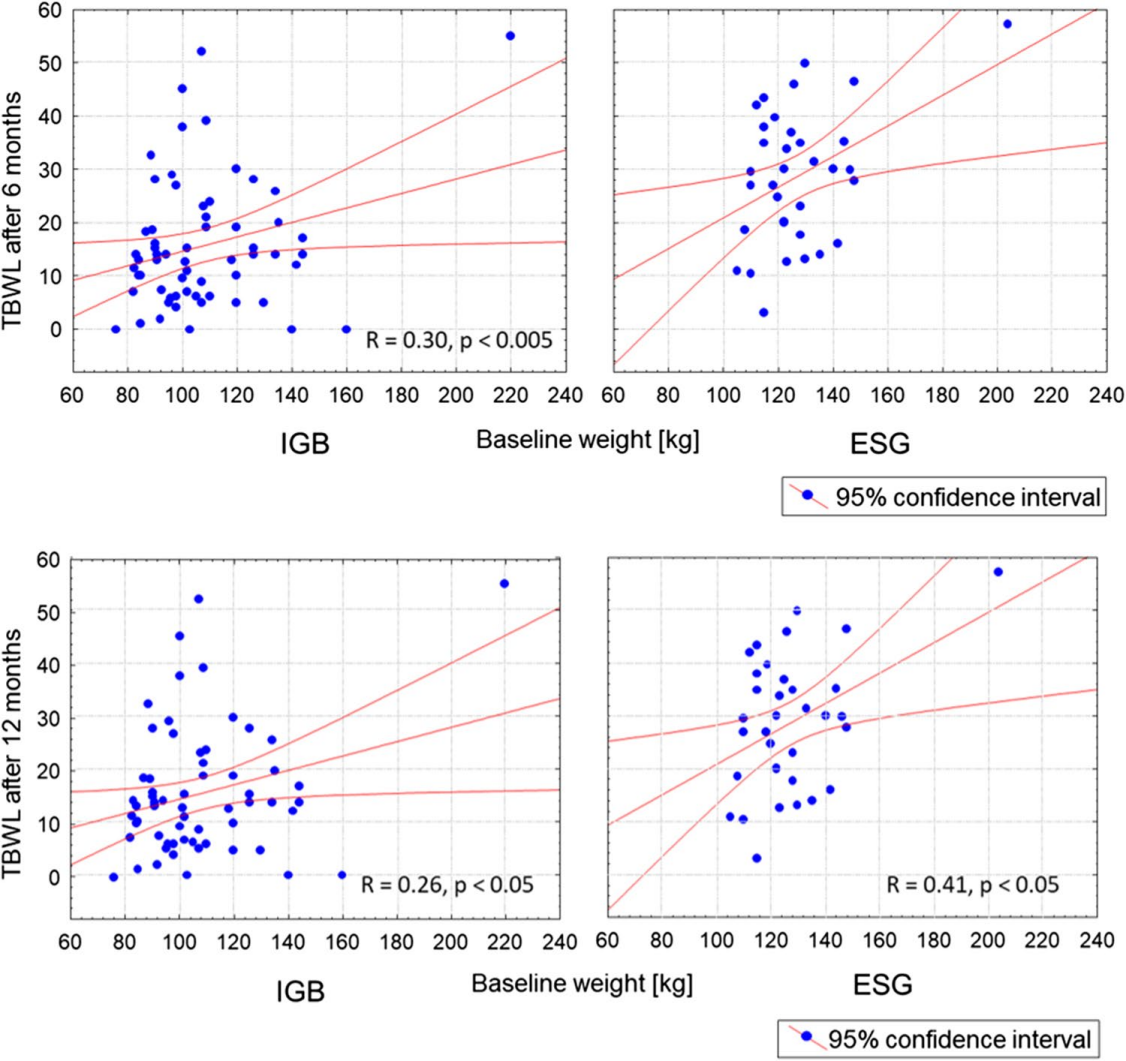
Correlation (*R*) between the total body weight loss (TBWL) and the baseline weight at 6 and 12 months after endoscopic sleeve gastrectomy (ESG) and intragastric balloon (IGB) placement

No differences in %TBWL were found between men and women when looking at the follow-up of either the ESG or IGB groups. Initial weight correlated with TBWL (higher baseline weight predisposed to more significant absolute weight loss in each group, *P* < 0.005) (Fig. 5), but did not correlate with %TBWL.

A detailed analysis of adverse events has not been performed. Nineteen out of 185 (10.27%) patients who had undergone IGB placement required earlier device removal due to intolerance manifested as nausea, vomiting, or abdominal pain. No AE were registered in the group treated with ESG. Neither deaths nor serious adverse events requiring surgical intervention have occurred..

## Discussion

Minimally invasive EBTs like IGB insertion or ESG have gained popularity over the last few years. Despite data confirming their efficacy and safety in general (5,8,10,11), there have been only a few studies comparing both of these EBT techniques [12–14]. Also, to our knowledge, there is no head-to-head comparison of IGB placement with different dwelling times (6 versus 12 months), nor the comparison of IGB placed for 12 months and ESG has been published. We evaluated the weight loss outcome of three EBT techniques: IGB with a dwelling time of 6 months, IGB with a dwelling time of 12 months, and ESG.

All procedures achieved good outcome taking into account the standards of recently published studies (%TBWL > 10%). However, ESG demonstrated the highest weight loss in comparison to both IGBs variants. In our study, a 6-month and 12-month %TBWL of 19.8% and 22.5% in the ESG group were consistent with both the values of 15.1% and 16.5% reported in a meta-analysis by Hedjoudje et al. [12], as well as 15.3% and 16.1% presented in meta-analysis by de Miranda Neto et al. [13]. Final weight loss achieved by both IGB modalities did not differ significantly. Our 6-month IGB group’s %TBWL of 15.3% was comparable with the 13.16% reported in a meta-analysis by Dayyeh et al. [8] and similar to our 12-month IGB group’s %TBWL of 14.7%. Alfredo et al. [14] previously reported consistent results proving no significant benefit of greater weight loss for balloons implanted for a up to 15 months versus 6 months. In a recently published prospective study evaluating the new Orbera365 including 97 patients, the authors reported a %TBWL of 16.2% in comparison to 15.5% seen after 6 months of device placement (*P* < 0.005) [9].

Our results indicate that weight loss slowed down after 6^th^ month following ESG and completely stopped for IGB patients. Although we measured only final outcome at IGB removal in the sixth or twelfth month, we presume that no weight loss occurred between the 6^th^ and 12^th^ month as the final results are comparable. This phenomenon may be explained by the stomach volume increasing over time, which allowed larger food intake. The advantage of prolonged dwelling time of IGB might be rather in maintaining than in increasing the result, as rebound weight gain after balloon removal is an expected outcome [14, 15]. Unfortunately, there are not many studies on long-term follow-up after IGB removal available, but a difficulty in maintaining weight loss following treatment with an IGB is reported by the majority of patients. A cohort study on 500 patients with obesity with a mean (SD) baseline BMI of 44.49 kg/m^2^ (8.46) demonstrated weight recidivism from a mean (SD) BMI of 35.74 kg/m^2^ (7.94) at time of IGB removal to 38.82 kg/m^2^ (7.71) after 12 months and 40.89 kg/m^2^ (7.75) after 24 months [16].

On the contrary, ESG seems to provide more sustainable results [17] as described in a meta-analysis including 1772 patients with a reported TBWL of 16.5% at 12 months and 17.1% at 18–24 months after ESG [12]. The loss to follow-up in the ESG group after 12 months (8 out of 42, 20%) was comparable or even lower than in previous studies [18, 19].

Interesting results were demonstrated by Lopez-Nava et al. in a prospective study on factors predicting success at 1 year after EBTs (IGB placement and ESG) in 962 patients, concluding that weight loss is more dependent on adherence to follow-up rather than on procedure type [18]. Moreover, the authors isolated ESG as an independent factor predicting significantly better adherence to follow-up, other factors being a higher initial BMI, higher % weight loss at 1 month and a female gender. Our analysis indicated that a higher baseline weight correlated with a greater weight loss but it was not reflected in a greater percentage of total body weight loss. Also, gender and age did not affect the final outcomes.

We did not evaluate the detailed adverse events (AEs) but there were no serious AEs requiring surgical intervention nor was there mortality associated with either procedure. A 10% early removal rate was observed after IGB placement, mostly due to vomiting or abdominal pain. Fayad et al. in their study evaluating these two procedures reported fewer AEs in the ESG group in comparison to the IGB group (5.2% vs. 17%; *P* = 0.048) [15]. The largest meta-analysis on ESG vs. IGB placement demonstrated high rates of AEs in both IGB and ESG group (abdominal pain 32.5% vs. 50.65, nausea 55.09% vs. 32.31%, respectively), but only 5.92% of them required early balloon removal. In the ESG, serious AEs were established at 1.52% [19]. Although there is very limited data on Orbera365, Jamal et al. proved its safety on the group of 97 patients (14% early balloon removal, 2% pancreatitis, no other serious AEs) [9].

We believe that the advantage of this study is a comparison of three EBTs—IGB inserted for 6 months, Orbera365, and ESG in terms of efficacy. To our knowledge, data regarding similar appraisal are scarce. There is only one study assessing the efficacy of Orbera365 and only a few studies comparing ESG to IGB [9, 15, 18, 19].

In addition, a standardized pre- or postprocedural care program was applied in all study groups. Also, it seems worth mentioning that the same endoscopist performed all IGB procedures and the same, experienced endoscopist performed all ESG procedures, which eliminates potential technique-related bias.

Our study has several limitations. First is its retrospective nature and the lack of randomization. The latter mirrors the need to offer various treatment options to patients, taking into account their preferences and financial capabilities. Second, there may exist a selection bias, as the baseline BMIs between the group treated with ESG and with IGB differed. That can hinder the comparability of the procedures. Third, there was no head-to-head comparison with diet only or with other noninvasive techniques for weight loss.

And finally, the study lacks long-term follow-up. We performed no follow-up after 12 months in the group of patients treated with IGB inserted for 6 months, so technically, we did not compare the head-to-head weight loss at the same time point in both groups treated with IGB. We did, however, focus on the direct efficacy of these procedures at the moment of IGB removal. We assumed that weight recidivism affects all patients after IGB removal to an extent depending on adherence to diet and exercise programs. To evaluate how IGB dwelling time affected weight-loss maintenance after IGB removal, we would have to conduct a long-term follow-up in both groups. Nevertheless, we believe that we provided new information regarding effects of the Orbera365 compared to a standard 6-month IGB treatment as this is one of the earliest studies on this type of IGB and the first study comparing these methods. Obviously, more studies are needed to determine the Orbera365 safety and efficacy.

Future research should focus on long-term follow-up after EBTs, assessing the general health outcomes, establishing their cost-effectiveness, and examining their performance against conventional bariatric interventions, including lifestyle therapies, in a randomized fashion. In addition, factors influencing procedural success require further exploration.

## Conclusion

Our study showed that both ESG and IGB insertion are effective for achieving weight loss. ESG resulted in superior weight loss compared to IGB placement. There was no significant difference in 6-month and 12-month dwelling time for IGB outcomes.

## References

[1] World Health Organization. Obesity and overweight. Available at: https://www.who.int/news-room/fact-sheets/detail/obesityand-overweight. Accessed June 9, 2021.

[2] The Look AHEAD Research Group (2014). Eight-year weight losses with an intensive lifestyle intervention: the look AHEAD study. Obesity.

[3] Lari E, Burhamah W, Lari A, Alsaeed T, Al-Yaqout K, Al-Sabah S (2021). Intra-gastric balloons – the past, present and future. Ann Med Surg.

[4] Bazerbachi F, Vargas EJ, Abu Dayyeh BK (2019). Endoscopic bariatric therapy: a guide to the intragastric balloon. Am J Gastroenterol.

[5] Vantanasiri K, Matar R, Beran A, Jaruvongvanich V (2020). The efficacy and safety of a procedureless gastric balloon for weight loss: a systematic review and meta-analysis. Obes Surg.

[6] Jirapinyo P, Thompson CC (2017). Endoscopic bariatric and metabolic therapies: surgical analogues and mechanisms of action. Clin Gastroenterol Hepatol.

[7] Claudia Gollisch KS, Raddatz D (2020). Endoscopic intragastric balloon: a gimmick or a viable option for obesity?. Ann Transl Med.

[8] Abu Dayyeh BK, Kumar N, Edmundowicz SA, Jonnalagadda S, Larsen M, Sullivan S (2015). ASGE Bariatric Endoscopy Task Force systematic review and meta-analysis assessing the ASGE PIVI thresholds for adopting endoscopic bariatric therapies. Gastrointest Endosc.

[9] Jamal MH, Al-Kanawati N, El Abd R, Al-Haddad M, AlKhadher T, Hamshari F (2021). A study examining the Orbera365 intragastric balloon safety and effects on weight loss. Obes Surg..

[10] Yoon JY, Arau RT, The study group for endoscopic bariatric and metabolic therapies in the Korean Society of Gastrointestinal Endoscopy. The efficacy and safety of endoscopic sleeve gastroplasty as an alternative to laparoscopic sleeve gastrectomy. Clin Endosc. 2021;54(1):17–24. 10.5946/ce.2021.019 PMC7939770 33478194

[11] Marincola G, Gallo C, Hassan C, Sessa L, Raffaelli M, Costamagna G (2021). Laparoscopic sleeve gastrectomy versus endoscopic sleeve gastroplasty: a systematic review and meta-analysis. Endosc Int Open..

[12] Hedjoudje A, Abu Dayyeh BK, Cheskin LJ, Adam A, Neto MG, Badurdeen D (2020). Efficacy and safety of endoscopic sleeve gastroplasty: a systematic review and meta-analysis. Clin Gastroenterol Hepatol..

[13] de Miranda Neto AA, de Moura DTH, Ribeiro IB, Khan A, Singh S, da Ponte Neto AM (2020). Efficacy and safety of endoscopic sleeve gastroplasty at mid term in the management of overweight and obese patients: a systematic review and meta-analysis. Obes Surg..

[14] Alfredo G, Roberta M, Francesca F, Massimiliano C, Pietro F, Daniela DP (2015). Intragastric balloon for obesity treatment: results of a multicentric evaluation for balloons left in place for more than 6 months. Surg Endosc.

[15] Fayad L, Cheskin LJ, Adam A, Badurdeen DS, Hill C, Agnihotri A (2019). Endoscopic sleeve gastroplasty versus intragastric balloon insertion: efficacy, durability, and safety. Endoscopy.

[16] Kotzampassi K, Grosomanidis V, Papakostas P, Penna S, Eleftheriadis E (2012). 500 Intragastric balloons: what happens 5 years thereafter?. Obes Surg.

[17] Lopez-Nava G, Sharaiha RZ, Vargas EJ, Bazerbachi F, Manoel GN, Bautista-Castaño I (2017). Endoscopic sleeve gastroplasty for obesity: a multicenter study of 248 patients with 24 months follow-up. Obes Surg.

[18] Lopez-Nava G, Asokkumar R, Rull A, Corbelle F, Beltran L, Bautista I (2019). Bariatric endoscopy procedure type or follow-up: what predicted success at 1 year in 962 obese patients?. Endosc Int Open.

[19] Singh S, de Moura DTH, Khan A, Bilal M, Chowdhry M, Ryan MB (2020). Intragastric balloon versus endoscopic sleeve gastroplasty for the treatment of obesity: a systematic review and meta-analysis. Obes Surg..

